# The AP-1 factor JUNB correlates with poor survival of patients with esophageal adenocarcinoma

**DOI:** 10.1038/s41598-025-07393-9

**Published:** 2025-07-23

**Authors:** Nikolai Schleussner, Karl Knipper, Ella Leugner, Christian Goddemeier, Uraz Yasar, Naita M. Wirsik, Jin-On Jung, Hans F. Fuchs, Lars M. Schiffmann, Alexander Quaas, Christiane J. Bruns, Thomas Schmidt

**Affiliations:** 1 Department of General, Visceral, Thorax and Transplantation Surgery, Faculty of Medicine and University Hospital Cologne, University of Cologne, Kerpener Str. 62, 50937 Cologne, Germany; 2 Department of General, Visceral and Transplantation Surgery, Faculty of Medicine and University Hospital Heidelberg, University of Heidelberg, Heidelberg, Germany; 3 Institute of Pathology, Faculty of Medicine and University Hospital Cologne, University of Cologne, Cologne, Germany

**Keywords:** Esophageal adenocarcinoma, AP-1, CJUN, JUNB, Targeted therapy, Individualized medicine, Cancer, Biomarkers, Molecular medicine, Oncology

## Abstract

Malignant cells have in contrast to non-transformed cells de-regulated transcriptional networks. The activator protein-1 (AP-1) transcription factor complex is expressed in many cancer entities including adenocarcinomas and has been correlated to de-regulated transcription and tumor-promoting mechanisms. Despite complex treatment approaches, esophageal cancer is still associated with poor overall survival. There is an urgent need for better patient stratification to increase the outcome of the multimodal treatment. This study investigated the expression of two AP-1 factors, cJUN and JUNB, and their role in 735 patients with esophageal cancer undergoing surgery. We performed immunohistochemical stainings for cJUN and JUNB and correlated the expression to the clinical outcome. Patients with a high JUNB expression level correlate to a reduced overall survival (OS) compared to patients with a low expression. Furthermore, in the multivariate analysis high JUNB expression was shown to be an independent risk factor for reduced patient survival. In addition, subgroup analysis demonstrated a significantly reduced OS for high JUNB expression in the subgroup of patients with neoadjuvant treatment. Strikingly, tumors co-expressing cJUN and JUNB were associated with poorer overall survival compared to those expressing only one or neither of the transcriptions factors. Our study suggests JUN expression as a novel biomarker to stratify patients, especially in the subgroup of neoadjuvant treated patients. Our findings have translational implications as targeting JUN might complement current available multimodal treatment approaches.

## Introduction

Esophageal adenocarcinoma (EA) is a highly aggressive tumor disease with a 5-year survival rate of only 22% and only 6% when metastases are already present at the time of diagnosis^1,2^. There are more than 550.000 new cases of esophageal cancer per year worldwide^3^. In Western industrialized nations, the incidence of EA is steadily increasing^4^. The only treatment options with a chance of cure are surgical resection or a definitive radiochemotherapy. The current standard of care for advanced tumors is a multimodal therapy with either a perioperative chemotherapy or neoadjuvant radiochemotherapy^5^. Despite all efforts, patients in an advanced stage have still a limited survival. The reasons for that are distant metastases, non-resectable locally advanced tumors, or treatment failure due to resistance to chemotherapy. The mechanisms, through which tumor cells achieve therapy resistance are not fully understood. However, distinct transcription factor programs are supposed to play a crucial role in this process^6,7^.

Among the transcription factors likely involved in the process of therapy resistance is AP-1^8^. AP-1 is a transcription factor complex composed of members from the multigenic JUN, FOS, ATF, and MAF families. The proteins of these families are basic leucine zipper proteins that form homo- or heterodimers^9^. They are ubiquitously expressed and have a wide range of functions. On the one hand, they activate the transcription of genes responsible for cell proliferation and differentiation, on the other hand, they also promote apoptosis and senescence^10^. AP-1 has already been shown to have proto-oncogenic effects in various tumor entities. They were shown to be relevant in the development of colorectal cancer (CRC), where a growth-promoting role was demonstrated for cJUN^11^. In oral and head and neck cancer AP-1 was correlated to poor patient survival^12^. In prostate cancer, JUN mediates senescence and acts as a tumor suppressor^13^.

Some AP-1 factors also seem to be relevant for EA, as AP-1 was correlated to chromatin remodeling and regulation of specific transcriptional programs in EA cells^14^. Furthermore, AP-1 acts downstream of ERBB2 in EA cells to control cell migration and adhesion^15^. However, the role of AP-1 in therapy resistance and cellular plasticity of esophageal cancer is still largely unclear.

As there is a desperate need to stratify patients into treatment responders and non-responders, this study aims to decipher the role of AP-1 in esophageal adenocarcinoma.

## Material and methods

### Patient samples

Seven hundred thirty-five patients with esophageal adenocarcinoma were included in this study, all underwent surgery in a curative intent at the University Hospital of Cologne between the years 1996 and 2019. Clinicopathological data were prospectively collected and retrospectively reviewed. Informed consent was obtained from all participants. Overall survival was defined as the time from surgery to either death or loss to follow-up. Patients who died or were lost to follow-up within 30 days of surgery were excluded from the analysis. As no cancer cells were available to assess JUN expression, patients who achieved a pathological complete response following neoadjuvant therapy were excluded from the analysis. The study was conducted in accordance with the principles of the Declaration of Helsinki and received approval from the University of Cologne’s ethics committee.

### Immunohistochemistry (IHC)

Tissue cylinders, 1.2 mm thick, were extracted from tumor samples using a semi-automated device and embedded in paraffin blocks, as previously described. Immunohistochemical staining for cJUN and JUNB was carried out using the Leica Bond-MAX automated system (Leica Biosystems, Wetzlar, Germany). cJUN (cJUN (60A8) Rabbit mAB #9165, Cell Signaling Technologies, 1:250 dilution) and JUNB (JUNB (C37F9) Rabbit mAB #3753, Cell Signaling Technologies, 1:250 dilution) staining was performed. An experienced pathologist (AQ) evaluated the staining intensity semi-quantitatively. Tumors were categorized according to the intensity (no staining = 0, low intensity = 1, high intensity = 2) and the number of positive cells (under 10% of the cells = 1, 10–20% = 2; 21–50% = 3, 51–75% = 4 and 76–100% = 5). Out of this information a modified H-score was calculated (0–10). Included patients were categorized as either low expression or high expression for each staining. All tumors with a modified H-score below 5 were classified as low expression and all tumors with a modified H-score of 5 or higher were classified as high expression.

### Statistics

Statistical analyses were conducted using IBM SPSS Statistics (Version 29.0.1.1; Armonk, USA). A p-value of less than 0.05 was considered statistically significant. The Chi-Square test was used to evaluate qualitative variables. Survival analyses were performed using Kaplan–Meier curves, with comparisons made via the log-rank test. Univariate and multivariate Cox regression analyses were employed to identify relationships between clinicopathological factors and survival outcomes. Only clinicopathological variables with a p-value below 0.20 in univariate cox regression were included in the multivariate analysis. All clinical data were prospectively collected and retrospectively analyzed. Overall survival was calculated from the date of surgery to either death or loss of follow-up.

## Results

735 patients with the diagnosis of an esophageal adenocarcinoma receiving curative intended surgery in the University Hospital of Cologne were included in this study. The clinicopathological characteristics are shown in Table 1. The majority of the patients were male (88% vs. 12% female). The median age was 66 years. Over 60% of all patients had a local (y)pT3 stage and in over 60% of the cases, at least one lymph node was positive (y)pN +. 505 out of 735 patients received a neoadjuvant or perioperative (radio-)chemotherapy before surgery.

**Table 1:** Patients´ characteristics of the total population as well as JUNB and cJUN negative and positive groups.

**Characteristic**	**Total**	**JUNB**		**p-value**	**cJUN**		**p-value**
		**negative**	**positive**		**negative**	**positive**	
	**n (%)**	**n (%)**	**n (%)**		**n (%)**	**n (%)**	
**No. of patients**	735 (100)	529 (100)	170 (100)		480 (100)	208 (100)	
**Sex**				**0.019**			0.331
Male	647 (88.0)	456 (86.2)	158 (92.9)		419 (87.3)	187 (89.9)	
Female	88 (12.0)	73 (13.8)	12 (7.1)		61 (12.7)	21 (10.1)	
**Age**				0.675			0.466
< 65	403 (54.8)	289 (54.6)	96 (56.5)		266 (55.4)	109 (52.4)	
> 65	332 (45.2)	240 (45.4)	74 (43.5)		214 (44.6)	99 (47.6)	
**Median overall survival (months)**	22.5	24.1	15.0		22.8	21.4	
**(Minimum–Maximum)**	(1.0–233.6)	(1.0–225.8)	(1.1–233.6)		(1.0–225.8)	(1.2–233.6)	
**Perioperative/** **neoadjuvant therapy**				0.180			0.246
No	230 (31.3)	162 (30.6)	55 (32.4)		160 (33.3)	60 (28.8)	
Yes	505 (68.7)	367 (69.4)	115 (67.6)		320 (66.7)	148 (71.2)	
**Regression grade**				**0.033**			**0.005**
1	216 (50.1)	156 (50.2)	52 (53.6)		156 (57.6)	51 (41.1)	
2	164 (38.1)	129 (41.2)	28 (28.9)		87 (32.1)	56 (45.2)	
3	49 (11.4)	26 (8.3)	16 (16.5)		28 (10.3)	15 (12.1)	
4	2 (0.5)	1 (0.3)	1 (1.0)		0 (0)	2 (1.6)	
**Response**				**0.013**			0.327
Minor	380 (88.2)	286 (91.4)	80 (82.5)		243 (89.7)	107 (86.3)	
Maior/complete	51 (11.8)	27 (8.6)	17 (17.5)		28 (10.3)	17 (13.7)	
**(y)pT**				0.556			0.133
1	131 (17.8)	93 (17.6)	25 (14.7)		78 (16.3)	46 (22.1)	
2	121 (16.5)	90 (17.0)	24 (14.1)		83 (17.3)	30 (14.4)	
3	459 (62.4)	329 (62.2)	114 (67.1)		299 (62.3)	128 (61.5)	
4	24 (3.3)	17 (3.2)	7 (4.1)		20 (4.2)	4 (1.9)	
**(y)pN**				**0.008**			0.301
0	293 (39.9)	224 (42.3)	47 (27.6)		183 (38.1)	93 (44.7)	
1	218 (29.7)	150 (28.4)	60 (35.3)		148 (30.8)	55 (26.4)	
2	104 (14.1)	71 (13.4)	31 (18.2)		66 (13.8)	31 (14.9)	
3	120 (16.3)	84 (15.9)	32 (18.8)		83 (17.3)	29 (13.9)	
**L**				0.278			0.857
0	337 (45.8)	245 (46.3)	70 (41.2)		215 (44.8)	97 (46.6)	
1	279 (38.0)	197 (37.2)	75 (44.1)		184 (38.3)	79 (38.0)	
2	119 (16.2)	87 (16.4)	25 (14.7)		81 (16.9)	32 (15.4)	
**V**				**0.019**			0.871
0	545 (74.1)	399 (75.4)	121 (71.2)		355 (74.0)	154 (74.0)	
1	76 (10.3)	45 (8.5)	27 (15.9)		48 (10.0)	23 (11.1)	
2	114 (15.5)	85 (16.1)	22 (12.9)		77 (16.0)	31 (14.9)	
**Pn**				0.226			0.697
0	469 (63.8)	339 (64.1)	105 (61.8)		300 (62.5)	137 (65.9)	
1	156 (21.2)	107 (20.2)	44 (25.9)		105 (21.9)	42 (20.2)	
2	110 (15.0)	83 (15.7)	21 (12.4)		75 (15.6)	29 (13.9)	
**G**				0.022			0.200
1	1 (0.4)	1 (0.6)	0 (0)		0 (0)	1 (1.7)	
2	121 (53.5)	94 (58.8)	20 (37.7)		86 (55.1)	29 (48,3)	
3	104 (46.0)	65 (40.6)	33 (62.3)		70 (44.9)	30 (50.0)	
**PD-L1**				0.565			0.108
Negative	325 (85.2)	229 (85.8)	80 (83.3)		219 (83.6)	92 (90.2)	
Positive	56 (14.7)	38 (14.2)	16 (16.7)		43 (16.4)	10 (9.8)	
**HER2/neu**				0.576			0.145
Negative	587 (88.8)	419 (88.0)	139 (89.7)		388 (89.6)	166 (85.6)	
Positive	74 (11.2)	57 (12.0)	16 (10.3)		45 (10.4)	28 (14.4)	

**Fig. 1:**
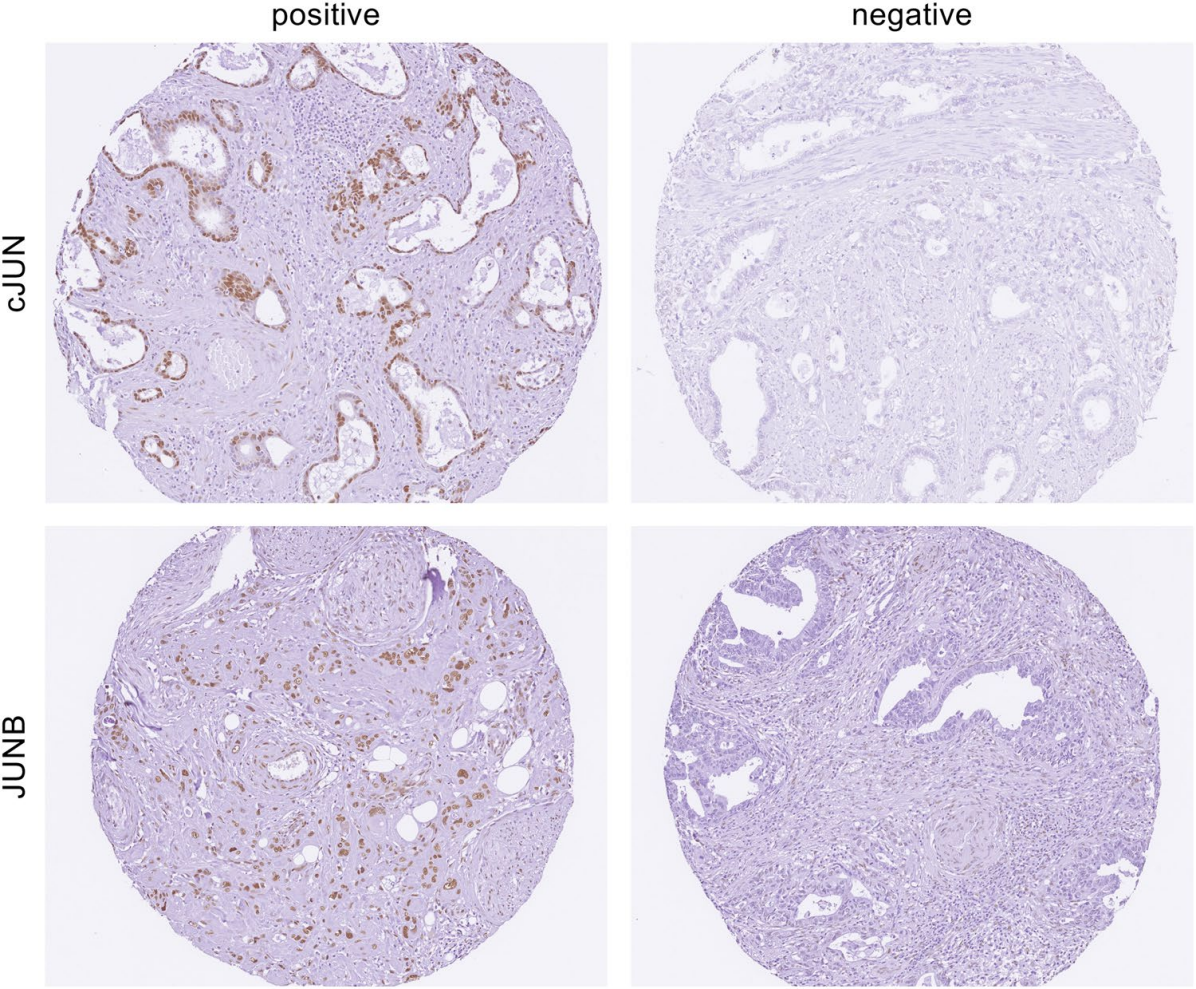
Immunohistochemistry (IHC) of cJUN and JUNB in esophageal adenocarcinoma. In total, 735 patient samples were stained for cJUN and JUNB. Representative immunohistochemistry pictures are shown: Top, cJUN IHC for a positive (left) and a negative sample (right). Bottom, JUNB IHC for a positive (left) and a negative sample (right). Magnification 20X.

In order to analyze the impact of the expression of cJUN and JUNB for esophageal cancer patients, we divided the patients based on their expression levels of the two transcription factors (cJUN: n = 208 positive; JUNB: n = 170 positive). JUNB-positive patients had higher pN-stages and higher vascular invasions. For cJUN-positive patients no such significance could be shown (Fig. 1 and Table 1).

**Fig. 2:**
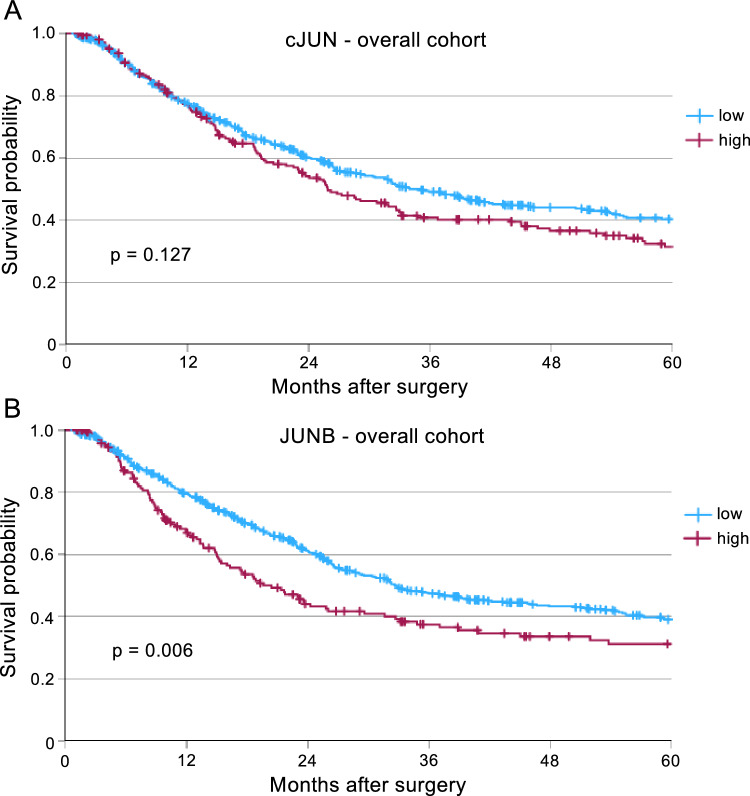
Expression of JUNB correlates to poor overall survival in esophageal adenocarcinoma. Kaplan-Meyer curves of the overall survival in the total cohort depending on (**A**) Low (n = 481) or high expression (n = 208) of cJUN (p = 0.127), and (**B**) low (n = 530) or high expression (n = 170) of JUNB are shown (p = 0.006).

To decipher the role of cJUN and JUNB on patient´s survival, we performed survival analyses and created Kaplan–Meier curves. Whereas for cJUN we could only detect a trend but no significance (p = 0.127) (Fig. 2A), high expression of JUNB correlated with an unfavorable patient survival (p = 0.006) in the overall cohort (Fig. 2B).

Following this, we performed univariate analyses for all baseline clinicopathological parameters and JUNB as well as cJUN staining. This showed higher age, neoadjuvant therapy, higher pT-, higher pN-stages, vascular invasion, tumor grade, regression, HER2/neu positivity as well as positive JUNB staining significantly correlated to poor overall survival (Table 2).

**Table 2:** Univariate cox regression of the total population. Bold print marks significant p-values (< 0.05).

Characteristic	Borders	Hazard Ratio	95% confidence interval	p—value
Sex	female vs male	0.753	0.547—1.036	0.081
Age	≥ 65 vs < 65	1.229	1.013–1.491	**0.036**
Perioperative/ neoadjuvant therapy	yes vs no	1.345	1.082–1.673	**0.008**
(y)pT	≥ 2 vs 1	1.625	1.425—1.853	** < 0.001**
(y)pN	≥ 1 vs 0	1.707	1.567—1.858	** < 0.001**
L	≥ 1 vs 0	1.056	0.936–1.193	0.375
V	≥ 1 vs 0	0.867	0.762–0.987	**0.030**
Pn	≥ 1 vs 0	0.958	0.848–1.083	0.494
G	≥ 1 vs 0	1.833	1.261–2.664	**0.002**
Regression grade	≥ 2 vs 1	0.782	0.650–0.940	**0.009**
Response	minor vs maior/complete	0.679	0.449–1.028	0.067
PD-L1	positive vs negative	0.872	0.604–1.261	0.467
Her2/neu	positive vs negative	0.559	0.386–0.808	**0.002**
JUNB	positive vs negative	1.371	1.095—1.716	**0.006**
cJUN	positive vs negative	1.024	0.937—1.118	0.602

Additionally, we performed multivariate Cox regression analyses in the overall cohort to detect any interdependencies. High JUNB expression was found to be an independent significant risk factor for poor overall survival in esophageal adenocarcinoma (hazard ratio, 1.397, 95% CI, 1.103–1.769; p = 0.005). Higher (y)pT- and (yp)N-stage, male sex and higher age were also shown to be independent risk factors (Table 3).

**Table 3:** Multivariate cox regression of the total population including JUNB expression. Bold print marks significant p-values (< 0.05). Variables with a p-value above 0.20 in the univariate cox regression (Table 2) were excluded for the multivariate analysis.

Characteristic	Borders	Hazard ratio	95% confidence interval	p—value
Sex	female vs male	0.675	0.480–0.948	**0.023**
Age	≥ 65 vs < 65	1.303	1.050—1.616	**0.016**
Perioperative/ neoadjuvant therapy	yes vs no	1.006	0.783—1.293	0.962
(y)pT	≥ 2 vs 1	1.315	1.119—1.546	** < 0.001**
(y)pN	≥ 1 vs 0	1.543	1.398—1.702	** < 0.001**
V	≥ 1 vs 0	0.907	0.783—1.051	0.193
HER2/neu	positive vs negative	0.764	0.524—1.114	0.162
JUNB	positive vs negative	1.397	1.103—1.769	**0.005**

**Fig. 3:**
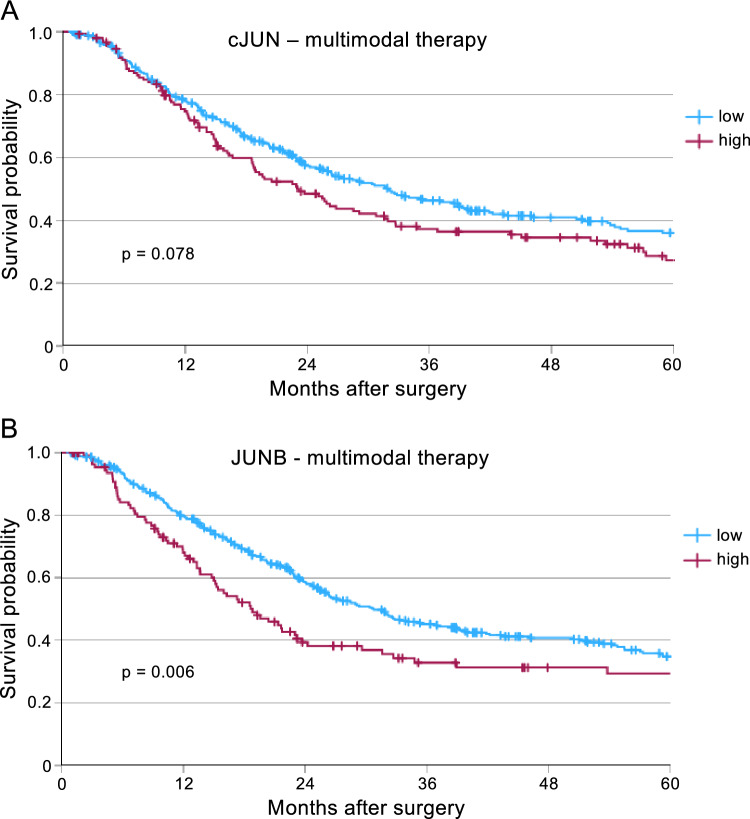
JUNB expression is a marker for poor outcome in patients with multimodal therapy. Survival analysis for the multimodally-treated patients using Kaplan-Meyer curves**.** (**A**) Low (n = 318) or high expression (n = 147) of cJUN (p = 0.078), and (**B**) low (n = 367) or high expression (n = 112) of JUNB is shown (p = 0.006).

Furthermore, subgroup analysis was conducted by stratifying patients based on their treatment strategy. In the primary surgery group – comprising patients who did not receive neoadjuvant treatment – no significant difference in overall survival was observed between those with low or high JUN expression (Supp. Figure 1). Interestingly, among patients who received neoadjuvant treatment prior to surgery, high cJUN expression showed a trend toward poorer overall survival, although this did not reach statistical significance (p = 0.078) (Fig. 3A). Furthermore, high JUNB expression correlated significantly with diminished overall survival (p = 0.006) (Fig. 3B).

**Fig. 4:**
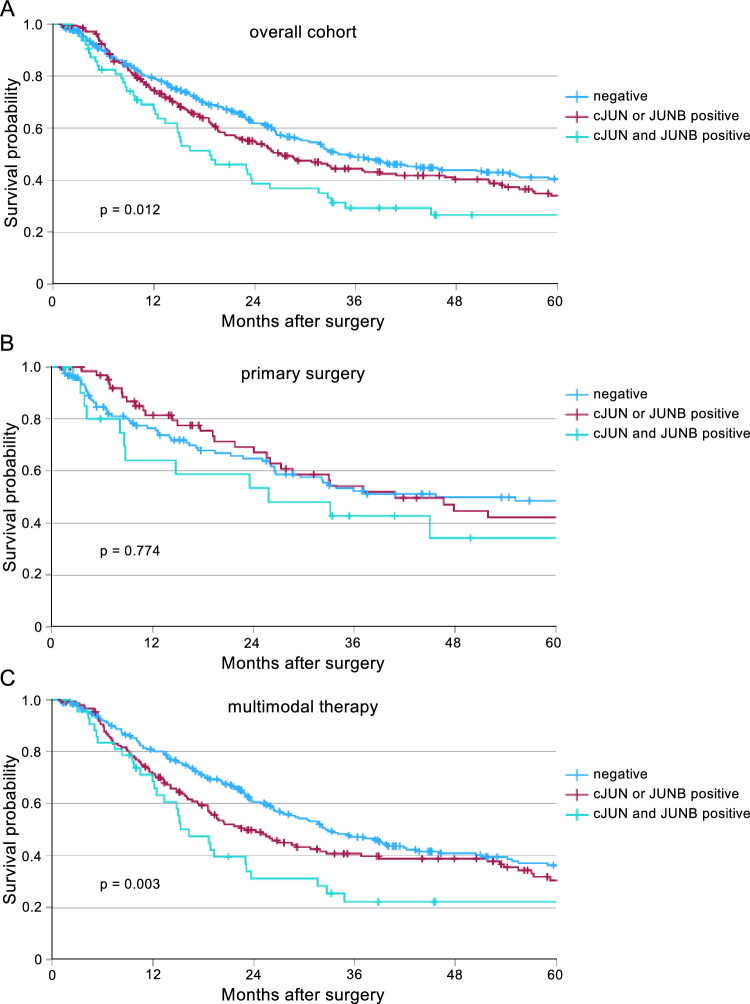
The combined expression of cJUN and JUNB correlates to the poorest outcome in esophageal adenocarcinoma. Survival analysis for the combined expression of cJUN and JUNB compared to either one of the two factors positive or none at all. (**A**) Overall cohort, negative (n = 370), cJUN or JUNB positive (n = 217) as well as cJUN and JUNB positive (n = 66) expression is shown (p = 0.012). (**B**) Primary surgery, negative (n = 123), cJUN or JUNB positive (n = 66) as well as cJUN and JUNB positive (n = 22) expression is shown (p = 0.774). (**C**) Multimodally-treated cohort, negative (n = 247), cJUN or JUNB positive (n = 44) as wells as cJUN and JUNB positive (n = 66) expression is shown (p = 0.003).

Given the overlapping functions of cJUN and JUNB, as well as their known ability to interact and to compensate for one another^16^, we analyzed the combined expression of cJUN to the expression of either factor alone or absence of both. The clinicopathological characteristics of these groups are shown in Supp. Table 1. Strikingly, in the overall cohort, patients co-expressing both proteins exhibited the poorest overall survival (p = 0.012) (Fig. 4A). The subgroup of primary surgery showed no significant difference in overall survival (p = 0.774) (Fig. 4B). However, in patients receiving neoadjuvant treatment, the combined expression of cJUN and JUNB correlated to a significantly diminished overall survival (p = 0.003) (Fig. 4C).

Furthermore, in the multivariate cox analysis the co-expression of cJUN and JUNB was found to be a significant risk factor for worse overall survival in esophageal adenocarcinoma (hazard ratio, 1.315, 95% CI, 1.131–1.530; p < 0.001). Higher (y)pT- (hazard ratio, 1.317, 95% CI, 1.117–1.552; p = 0.001) and (yp)N-stages (hazard ratio, 1.541, 95% CI, 1.392–1.706; p < 0.001) were also shown to be independent risk factors (Table 4 and Supp. Table 2).

**Table 4:** Multivariate cox regression of the total population including the combined expression of JUNB + cJUN. Bold print marks significant p-values (< 0.05). Variables with a p-value above 0.20 in the univariate cox regression (Supp Table 2) were excluded for the multivariate analysis.

Characteristic	Borders	Hazard ratio	95% confidence interval	p—value
Age	≥ 65 vs < 65	1.213	0.972—1.513	0.087
Perioperative/ neoadjuvant therapy	yes vs no	1.015	0.787—1.311	0.907
(y)pT	≥ 2 vs 1	1.317	1.117—1.552	**0.001**
(y)pN	≥ 1 vs 0	1.541	1.392—1.706	** < 0.001**
V	≥ 1 vs 0	0.911	0.783—1.060	0.229
HER2/neu	positive vs negative	0.723	0.492—1.062	0.099
JUNB + cJUN	positive vs negative	1.315	1.131—1.530	** < 0.001**

In summary, this study demonstrates that high JUNB expression is associated with reduced overall survival in the entire cohort, with this effect being particularly pronounced in the subgroup of patients who received neoadjuvant therapy. Furthermore, multivariate analysis identified high JUNB expression as an independent risk factor for poor survival outcomes. Strikingly, patients whose tumors co-expressed both cJUN and JUNB exhibited the worst overall survival, significantly lower than that observed in patients expressing only one or neither of these transcription factors.

Taken together, the data support a role for elevated AP-1 transcriptional activity in driving tumor progression and underscore the clinical relevance of JUNB and cJUN as prognostic markers in EAC.

## Discussion

Our study identified the AP-1 transcription factor JUNB as being associated with poor clinical outcomes in patients with esophageal adenocarcinoma. We showed that patients with high expression of JUNB correlated with a significantly reduced overall survival. Furthermore, JUNB was found to be an independent risk factor for poor overall outcome. Notably, the combined expression of cJUN and JUNB was associated with the worst overall survival.

AP-1 is a key player for multiple cellular functions^17^ and previous studies showed AP-1 factors being upregulated in many different solid cancers including esophageal squamous cell carcinoma^18^, non-small cell lung cancer^19^, pancreatic cancer^20^ colorectal cancer^11^, and non-solid malignancies for example lymphomas^21,22^. In the work presented here, high JUNB expression showed a worse overall survival. Given the evidence above, high expression of AP-1 in esophageal adenocarcinomas might lead to an increased proliferation and better survival of the cancer cells leading to a higher tumor burden. This may contribute to the poorer clinical outcomes of patients with high AP-1 activity. Supporting this, the downregulation of AP-1 transcription factors *in-vitro* leads to a diminished survival of tumor cells and upregulation of apoptosis^23^.

AP-1 activity was shown to control EMT-related processes like migration and adhesion in esophageal adenocarcinoma cell lines^15^ and other cancer entities such as glioblastoma, breast, and colorectal cancer^24–26^. EMT-associated transcriptional reprogramming facilitates cancer cell stemness^27^ and thereby enables the tumor cells to evade anticancer treatments^28^. In ovarian cancer cells the AP-1 member FRA-1 leads to reduced chemosensitivity to adriamycin and etoposide^29^.

In this study, the subgroup of patients who received neoadjuvant therapy exhibited the strongest correlation between AP-1 expression and poor clinical outcome. An explanation for this could either be an upregulation of JUNB under the course of the (neoadjuvant) treatment, or a survival advantage of tumor cells highly expressing JUNB. This might represent cancer stem cells leading to recurrence and metastasis. There is some evidence for a role of AP-1 factors in chemotherapy resistance: JUNB was shown to mediate oxaliplatin resistance in gastric cancer cells, by inducing DNA-repair-enzymes. Reduction of JUNB using shRNA reversed Oxaliplatin resistance and resulted in massive cell death^30^. A similar resistance process for cisplatin is observable in mesothelioma via deregulation of c-fos/AP-1 complex^31^. Elevated JUNB was additionally associated with therapy resistance in Multiple Myeloma. Knockdown of JunB restored the response to dexamethasone in dexamethasone-resistant cells^32^.

Additionally, a clinical pilot study researching the responsiveness of neoadjuvant chemotherapy in rectal adenocarcinoma found eight upregulated genes in non-responding patients. One of these significantly deregulated genes is JUNB^33^.

Further studies should address this topic and might reveal the JUN family as a potential target esophageal adenocarcinoma. There are indeed some promising results regarding AP-1 inhibition in cancer: Daisuke et al. could show that the AP-1 inhibitor T-5224 inhibits lymph node metastasis in head and neck cancer. Interestingly, the growth of the primary tumor was not affected^34^. Zhang et al. could further show that another AP-1 inhibitor SR11302 inhibited both the primary tumor growth and lymph node metastasis in head and neck squamous cell carcinoma^35^. Therefore, it would be interesting to investigate the effect of AP-1 inhibition in esophageal cancer.

This study has some constraints due to its retrospective character. However, the analysis of more than 700 patients with an esophageal adenocarcinoma provides a profound ground for our results. Evaluating JUN expression in primary biopsies before neoadjuvant therapy could provide valuable information for clinicians. This could lead to better stratification of patients prior treatment and allow adjustments or intensification of neoadjuvant therapy for tumors. Mechanistic experiments including loss-of-function (LOF) and gain-of-function (GOF) such as knock-down using shRNA or siRNA and overexpression of JUNB in EAC cell lines and organoids should clarify the pathophysiological mechanisms of JUNs, as well as their impact on (radio-) chemoresistance and patient survival in esophageal adenocarcinoma.

We hypothesis that inhibition of JUNB leads to reduced proliferation, invasion and resistance to therapy. However, further prospective and biomolecular studies are necessary to confirm that JUN-positive tumors represent a high-risk subgroup with low responsiveness to neoadjuvant treatment in esophageal adenocarcinoma.

## Conclusion

In this study we report the AP-1 factor JUNB and the double-positivity for cJUN and JUNB as a potential biological marker for patients with a poor overall survival, specifically in the context of multimodal therapy. This warrants further investigation of AP-1 in esophageal cancer with respect to the exact mode of action and its potential to complement perioperative or neoadjuvant (radio)chemotherapy.

## Supplementary Information

Supplementary Information.

## Data Availability

The datasets generated and analyzed during the current study are available from the corresponding author on reasonable request.
