# Supplementary material for: The AP-1 factor JUNB correlates with poor survival of patients with esophageal adenocarcinoma
Source: Sci Rep. 2025 Jul 23;15:26790. doi: 10.1038/s41598-025-07393-9 (PMC12287255; doi:10.1038/s41598-025-07393-9)
Supplement: Supplementary file 1 — Supplementary Information. [file 41598_2025_7393_MOESM1_ESM.pdf]

Supplementary Material:

The AP-1 factor JUNB correlates with poor survival of patients with esophageal adenocarcinoma

Nikolai Schleussner[1] *, Karl Knipper[1], Ella Leugner[2], Christian Goddemeier[1], Uraz Yasar[2], Naita M. Wirsik[1], Jin-On Jung[1], Hans F. Fuchs[1], Lars M. Schiffmann[1], Alexander Quaas[3], Christiane J. Bruns[1], Thomas Schmidt[1]

Supplementary Figure 1

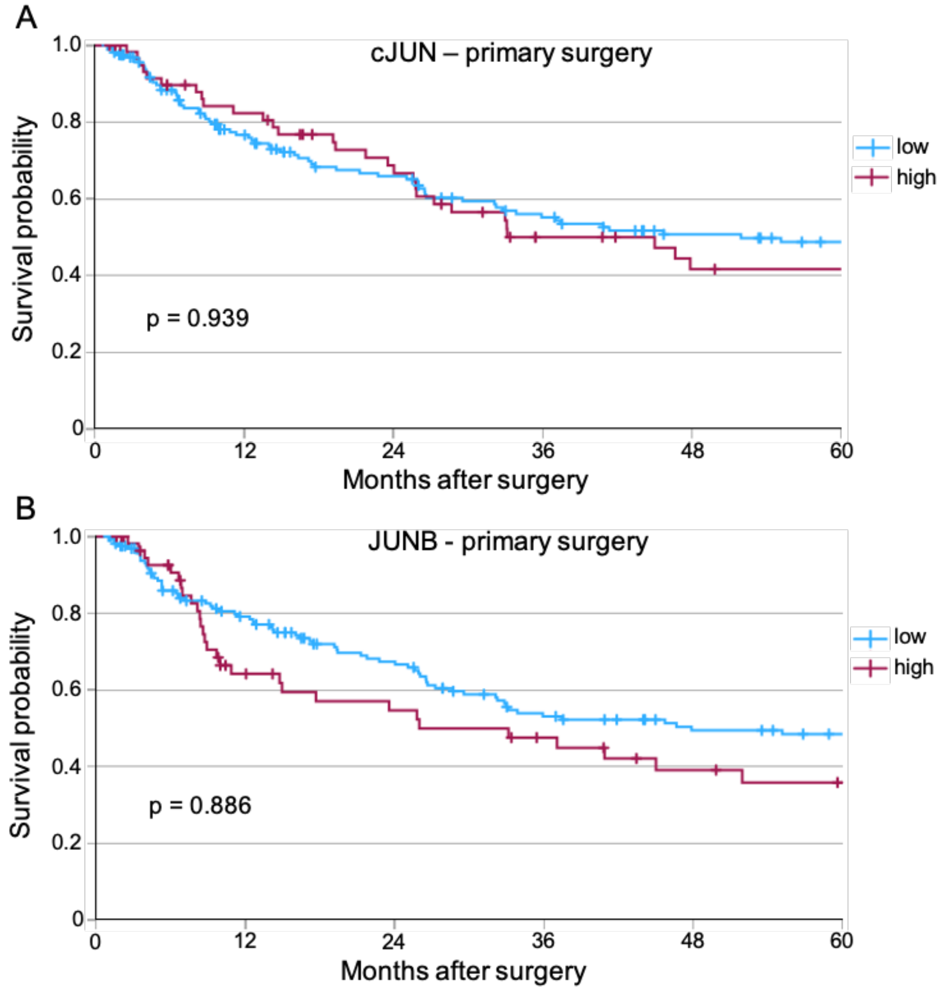

Supplementary Figure 1: Survival analysis for the patients with a primary operation. (A) Low (n = 163) or high expression (n = 61) of cJUN (p = 0.939), and (B) low (n = 163) or high expression (n = 58) expression of JUNB is shown (p = 0.346).

Supplementary Table 1:

Characteristic	Total<br>n (%)	negative<br>n (%)	cJUN or JUNB<br>n (%)	cJUN and JUNB<br>n (%)	p-value
**No. of patients**	653 (100)	370 (100)	217 (100)	66 (100)	
**Sex**					0.046
Male	574 (87.9)	315 (85.1)	199 (91.7)	60 (90.9)	
Female	79 (12.1)	55 (14.9)	18 (8.3)	6 (9.1)	
**Age**					0.461
< 65	358 (54.8)	202 (54.6)	124 (57.1)	32 (48.5)	
≥ 65	295 (45.2)	168 (45.4)	93 (42.9)	34 (51.5)	
**Median overall survival (months)**	22.5	23.7	19.6	14.9	
**(Minimum-Maximum)**	(1.0-233.6)	(1.0-225.8)	(1.1-202.2)	(1.6-233.6)	
**Perioperative/ neoadjuvant therapy**					0.765
No	211 (32.3)	123 (33.2)	66 (30.4)	22 (33.3)	
Yes	442 (67.7)	247 (66.8)	151 (69.6)	44 (66.7)	
**Regression grade**					0.074
1	200 (53.5)	115 (55.0)	72 (56.3)	13 (35.1)	
2	136 (36.4)	75 (35.9)	45 (35.2)	16 (43.2)	
3	36 (9.6)	19 (9.1)	10 (7.8)	7 (18.9)	
4	2 (0.5)	0 (0)	1 (0.8)	1 (2.7)	
**Response**					0.052
minor	336 (89.8)	190 (90.9)	117 (91.4)	29 (78.4)	
maior / complete	38 (10.2)	19 (9.1)	11 (8.6)	8 (21.6)	
**(y)pT**					0.711
1	111 (17.0)	60 (16.2)	40 (18.4)	11 (16.7)	
2	107 (16.4)	66 (17.8)	30 (13.8)	11 (16.7)	
3	411 (62.9)	228 (61.6)	142 (65.4)	41 (62.1)	
4	24 (3.7)	16 (4.3)	5 (2.3)	3 (4.5)	
**(y)pN**					0.455
0	254 (38.9)	147 (39.7)	88 (40.6)	19 (28.8)	
1	195 (29.9)	108 (29.2)	66 (30.4)	21 (31.8)	
2	95 (14.5)	51 (13.8)	29 (13.4)	15 (22.7)	
3	107 (16.4)	64 (17.3)	34 (15.7)	11 (16.7)	
**L**					0.741
0	291 (44.6)	171 (46.2)	93 (42.9)	27 (40.9)	
1	256 (39.2)	138 (37.3)	88 (40.6)	30 (45.5)	
2	106 (16.2)	61 (16.5)	36 (16.6)	9 (13.6)	
**V**					0.215
0	485 (74.3)	279 (75.4)	160 (73.7)	46 (69.7)	
1	67 (10.3)	32 (8.6)	23 (10.6)	12 (18.2)	
2	101 (15.5)	59 (15.9)	34 (15.7)	8 (12.1)	
**Pn**					0.779
0	413 (63.2)	237 (64.1)	133 (61.3)	43 (65.2)	
1	142 (21.7)	76 (20.5)	50 (23.0)	16 (24.2)	
2	98 (15.0)	57 (15.4)	34 (15.7)	7 (10.6)	
**G**					0.069
1	1 (0.5)	0 (0)	1 (1.6)	0 (0)	
2	108 (53.2)	70 (58.3)	32 (51.6)	6 (28.6)	
3	94 (46.3)	50 (41.7)	29 (46.8)	15 (71.4)	

**PD-L1**					0.700
negative	295 (85.3)	168 (84.8)	92 (84.4)	35 (89.7)	
positive	51 (14.7)	30 (15.2)	17 (15.6)	4 (10.3)	
**HER2/neu**					0.862
negative	525 (87.9)	296 (88.4)	174 (87.9)	55 (85.9)	
positive	72 (12.1)	39 (11.6)	24 (12.1)	9 (14.1)	

Supplementary Table 1: Patients´ characteristics of the total population as well as negative, JUNB or cJUN single positive and double positive groups.

Supplementary Table 2:

Characteristic	Borders	Hazard Ratio	95 % confidence interval	p - value
**Sex**	female vs male	0.805	0.577 - 1.122	0.200
**Age**	≥ 65 vs < 65	1.254	1.023 – 1.536	**0.029**
**Perioperative/ neoadjuvant therapy**	yes vs no	1.354	1.078 – 1.702	**0.009**
**(y)pT**	≥ 2 vs 1	1.668	1.451 - 1.918	**< 0.001**
**(y)pN**	≥ 1 vs 0	1.683	1.539 - 1.841	**< 0.001**
**L**	≥ 1 vs 0	1.029	0.905 – 1-171	0.661
**V**	≥ 1 vs 0	0.846	0.738 – 0.970	**0.016**
**Pn**	≥ 1 vs 0	0.936	0.823 – 1.066	0.321
**G**	≥ 1 vs 0	1.755	1.189 – 2.589	**0.005**
**Regression grade**	≥ 2 vs 1	0.828	0.677 – 1.012	0.066
**Response**	minor vs maior / complete	0.757	0.472 – 1.212	0.246
**PD-L1**	positive vs negative	0.840	0.571 – 1.235	0.375
**Her2/neu**	positive vs negative	0.540	0.371 – 0.786	**0.001**
**JUNB+cJUN**	positive vs negative	1.224	0.998 - 1.501	0.052

Supplementary Table 1: Univariate cox regression of the total population for co-expression of JUNB and cJUN. Bold print marks significant p-values (< 0.05) Variables with a p-value above 0.20 were excluded for the multivariate analysis.
